# Supplementary material for: Cannabis Seedlings Inherit Seed-Borne Bioactive and Anti-Fungal Endophytic Bacilli
Source: Plants (Basel). 2022 Aug 15;11(16):2127. doi: 10.3390/plants11162127 (PMC9415172; doi:10.3390/plants11162127)
Supplement: Supplementary file 1 [file plants-11-02127-s001.zip › plants-1862324-supplementary.pdf]

plants

MDPI

Table S1. Isolate Metadata.

Sample ID	Taxonomy	Kingdom	Host	Genotype	Tissue	Media	Protocol	Surface sterilization
**OKM58**	*Paenibacillus mobilis*	Bacteria	Hemp	Canda	Seedling	TSA	Maceration	Bleach+EtOH
**OKM59**	*Pantoea agglomerans*	Bacteria	Hemp	Canda	Seedling	TSA	Maceration	Bleach+EtOH
**OKM60**	*Paenibacillus mobilis*	Bacteria	Hemp	Canda	Seedling	TSA	Maceration	Bleach+EtOH
**OKM62**	*Paenibacillus mobilis*	Bacteria	Hemp	Canda	Seedling	TSA	Maceration	Bleach+EtOH
**OKM63**	*Paenibacillus mobilis*	Bacteria	Hemp	Canda	Seedling	TSA	Maceration	Bleach+EtOH
**OKM64**	*Paenibacillus mobilis*	Bacteria	Hemp	Canda	Seedling	R2A	Maceration	Bleach+EtOH
**OKM65**	*Paenibacillus mobilis*	Bacteria	Hemp	Canda	Seedling	R2A	Maceration	Bleach+EtOH
**OKM67**	*Paenibacillus mobilis*	Bacteria	Hemp	Canda	Seedling	R2A	Maceration	Bleach+EtOH
**OKM68**	*Paenibacillus mobilis*	Bacteria	Hemp	Canda	Seedling	R2A	Maceration	Bleach+EtOH
**OKM69**	*Paenibacillus mobilis*	Bacteria	Hemp	Canda	Seedling	R2A	Maceration	Bleach+EtOH
**OKM76**	*Paenibacillus mobilis*	Bacteria	Marijuana	White Widow	Seedling	R2A	Maceration	Bleach+EtOH
**OKM77**	*Paenibacillus illinoisensis*	Bacteria	Marijuana	White Widow	Seedling	R2A	Maceration	Bleach+EtOH
**OKM78**	*Bacillus megaterium*	Bacteria	Marijuana	White Widow	Seedling	R2A	Maceration	Bleach+EtOH
**OKM79**	*Paenibacillus illinoisensis*	Bacteria	Marijuana	White Widow	Seedling	R2A	Maceration	Bleach+EtOH
**OKM80**	*Paenibacillus illinoisensis*	Bacteria	Marijuana	White Widow	Seedling	R2A	Maceration	Bleach+EtOH
**OKM81**	*Paenibacillus illinoisensis*	Bacteria	Marijuana	White Widow	Seedling	R2A	Maceration	Bleach+EtOH
**OKM82**	*Paenibacillus illinoisensis*	Bacteria	Marijuana	White Widow	Seedling	R2A	Maceration	Bleach+EtOH
**OKM83**	*Paenibacillus illinoisensis*	Bacteria	Marijuana	Afghani	Seedling	TSA	Maceration	Bleach+EtOH
**OKM84**	*Paenibacillus taohuashanense*	Bacteria	Marijuana	Afghani	Seedling	TSA	Maceration	Bleach+EtOH
**OKM85**	*Paenibacillus pabuli*	Bacteria	Marijuana	Afghani	Seedling	TSA	Maceration	Bleach+EtOH
**OKM86**	*Paenibacillus pabuli*	Bacteria	Marijuana	Afghani	Seedling	TSA	Maceration	Bleach+EtOH
**OKM87**	*Paenibacillus mobilis*	Bacteria	Marijuana	Afghani	Seedling	TSA	Maceration	Bleach+EtOH
**OKM88**	*Paenibacillus pabuli*	Bacteria	Marijuana	White Widow	Seedling	TSA	Maceration	Bleach+EtOH
**OKM89**	*Paenibacillus mobilis*	Bacteria	Marijuana	White Widow	Seedling	TSA	Maceration	Bleach+EtOH
**OKM90**	*Paenibacillus pabuli*	Bacteria	Marijuana	God's Pink	Seedling	R2A	Maceration	Bleach+EtOH
**OKM92**	*Paenibacillus mobilis*	Bacteria	Marijuana	God's Pink	Seedling	R2A	Maceration	Bleach+EtOH
**OKM93**	*Paenibacillus mobilis*	Bacteria	Marijuana	God's Pink	Seedling	TSA	Maceration	Bleach+EtOH
**OKM94**	*Bacillus megaterium*	Bacteria	Marijuana	God's Pink	Seedling	R2A	Maceration	Bleach+EtOH
**OKM95**	*Bacillus/Peribacillus simplex*	Bacteria	Marijuana	BC Big Bud	Seedling	R2A	Maceration	Bleach+EtOH
**OKM96**	*Bacillus inaquosorum*	Bacteria	Marijuana	BC Big Bud	Seedling	TSA	Maceration	Bleach+EtOH
**OKM97**	*Bacillus aerius*	Bacteria	Marijuana	BC Big Bud	Seedling	TSA	Maceration	Bleach+EtOH
**OKM98**	*Bacillus stratosphericus*	Bacteria	Marijuana	BC Big Bud	Seedling	TSA	Maceration	Bleach+EtOH
**OKM99**	*Paenibacillus mobilis*	Bacteria	Marijuana	BC Big Bud	Seedling	TSA	Maceration	Bleach+EtOH
**OKM100**	*Bacillus aerius*	Bacteria	Marijuana	BC Big Bud	Seedling	TSA	Maceration	Bleach+EtOH
**OKM101**	*Paenibacillus mobilis*	Bacteria	Marijuana	BC Big Bud	Seedling	TSA	Maceration	Bleach+EtOH
**OKM102**	*Paenibacillus pabuli*	Bacteria	Marijuana	BC Big Bud	Seedling	TSA	Maceration	Bleach+EtOH
**OKM103**	*Paenibacillus mobilis*	Bacteria	Hemp	X-59	Seedling	TSA	Maceration	Bleach+EtOH
**OKM104**	*Bacillus circulans*	Bacteria	Hemp	X-59	Seedling	TSA	Maceration	Bleach+EtOH
**OKM105**	*Paenibacillus vulneris*	Bacteria	Hemp	X-59	Seedling	TSA	Maceration	Bleach+EtOH
**OKM106**	*Bacillus megaterium*	Bacteria	Hemp	X-59	Seedling	R2A	Maceration	Bleach+EtOH
**OKM107**	*Paenibacillus mobilis*	Bacteria	Hemp	X-59	Seedling	R2A	Maceration	Bleach+EtOH
**OKM108**	*Brevibacillus choshinensis*	Bacteria	Marijuana	BC Big Bud	Seedling	R2A	Maceration	Bleach+EtOH
**OKM109**	*Paenibacillus taohuashanense*	Bacteria	Marijuana	BC Big Bud	Seedling	R2A	Maceration	Bleach+EtOH
**OKM110**	*Paenibacillus mobilis*	Bacteria	Marijuana	BC Big Bud	Seedling	R2A	Maceration	Bleach+EtOH
**OKM111**	*Paenibacillus pabuli*	Bacteria	Marijuana	BC Big Bud	Seedling	R2A	Maceration	Bleach+EtOH
**OKM112**	*Bacillus megaterium*	Bacteria	Hemp	X-59	Seedling	TSA	Maceration	Bleach+EtOH
**OKM113**	*Bacillus megaterium*	Bacteria	Hemp	X-59	Seedling	TSA	Maceration	Bleach+EtOH
**OKM114**	*Paenibacillus mobilis*	Bacteria	Hemp	X-59	Seedling	R2A	Maceration	Bleach+EtOH
**OKM115**	*Bacillus subtilis*	Bacteria	Marijuana	BC Big Bud	Seedling	R2A	Maceration	Bleach+EtOH
**OKM116**	*Paenibacillus mobilis*	Bacteria	Marijuana	BC Big Bud	Seedling	R2A	Maceration	Bleach+EtOH
**OKM117**	*Brevibacillus choshinensis*	Bacteria	Marijuana	BC Big Bud	Seedling	R2A	Maceration	Bleach+EtOH
**OKM118**	*Paenibacillus mobilis*	Bacteria	Marijuana	God's Bud	Seedling	R2A	Maceration	Bleach+EtOH
**OKM119**	*Bacillus/Peribacillus simplex*	Bacteria	Marijuana	God's Bud	Seedling	R2A	Maceration	Bleach+EtOH

**OKM120**	*Paenibacillus mobilis*	Bacteria	Marijuana	God's Bud	Seedling	R2A	Maceration	Bleach+EtOH
**OKM121**	*Bacillus subtilis*	Bacteria	Marijuana	God's Bud	Seedling	TSA	Maceration	Bleach+EtOH
**OKM122**	*Paenibacillus mobilis*	Bacteria	Marijuana	God's Bud	Seedling	TSA	Maceration	Bleach+EtOH
**OKM123**	*Brevibacillus choshinensis*	Bacteria	Marijuana	God's Bud	Seedling	TSA	Maceration	Bleach+EtOH
**OKM124**	*Paenibacillus mobilis*	Bacteria	Marijuana	BC Big Bud	Seedling	R2A	Maceration	Bleach+EtOH
**OKM125**	*Bacillus/Peribacillus simplex*	Bacteria	Marijuana	BC Big Bud	Seedling	R2A	Maceration	Bleach+EtOH
**OKM126**	*Paenibacillus mobilis*	Bacteria	Marijuana	God's Bud	Seedling	TSA	Maceration	Bleach+EtOH
**OKM127**	*Paenibacillus mobilis*	Bacteria	Hemp	CAN 3797	Seedling	R2A	Maceration	Bleach+EtOH
**OKM128**	*Bacillus subtilis*	Bacteria	Hemp	CAN 3797	Seedling	R2A	Maceration	Bleach+EtOH
**OKM129**	*Paenibacillus mobilis*	Bacteria	Hemp	CAN 3797	Seedling	R2A	Maceration	Bleach+EtOH
**OKM130**	*Paenibacillus taohuashanense*	Bacteria	Hemp	CAN 3797	Seedling	R2A	Maceration	Bleach+EtOH
**OKM131**	*Bacillus subtilis*	Bacteria	Hemp	CAN 3797	Seedling	R2A	Maceration	Bleach+EtOH
**OKM132**	*Bacillus megaterium*	Bacteria	Hemp	CAN 3797	Seedling	R2A	Maceration	Bleach+EtOH
**OKM133**	*Bacillus megaterium*	Bacteria	Hemp	CAN 3797	Seedling	TSA	Maceration	Bleach+EtOH
**OKM134**	*Paenibacillus vulneris*	Bacteria	Hemp	CAN 3797	Seedling	TSA	Maceration	Bleach+EtOH
**OKM135**	*Paenibacillus mobilis*	Bacteria	Hemp	CAN 3797	Seedling	TSA	Maceration	Bleach+EtOH
**OKM136**	*Paenibacillus pabuli*	Bacteria	Hemp	CAN 3797	Seedling	TSA	Maceration	Bleach+EtOH
**OKM137**	*Paenibacillus mobilis*	Bacteria	Hemp	CAN 3797	Seedling	TSA	Maceration	Bleach+EtOH
**OKM138**	*Paenibacillus mobilis*	Bacteria	Hemp	CAN 3797	Seedling	TSA	Maceration	Bleach+EtOH
**OKM139**	*Paenibacillus mobilis*	Bacteria	Hemp	CAN 3797	Seedling	TSA	Maceration	Bleach+EtOH
**OKM140**	*Paenibacillus mobilis*	Bacteria	Hemp	CAN 3797	Seedling	TSA	Maceration	Bleach+EtOH
**OKM141**	*Bacillus velezensis*	Bacteria	Hemp	CAN 3797	Seedling	TSA	Maceration	Bleach+EtOH
**OKM142**	*Paenibacillus taohuashanense*	Bacteria	Hemp	CAN 3797	Seedling	TSA	Maceration	Bleach+EtOH
**OKM143**	*Bacillus subtilis*	Bacteria	Hemp	CAN 3797	Seedling	TSA	Maceration	Bleach+EtOH
**OKM144**	*Paenibacillus mobilis*	Bacteria	Hemp	CAN 3797	Seedling	TSA	Maceration	Bleach+EtOH
**OKM145**	*Paenibacillus mobilis*	Bacteria	Hemp	Grandi	Seedling	R2A	Maceration	Bleach+EtOH
**OKM146**	*Paenibacillus pabuli*	Bacteria	Hemp	Altair	Seedling	TSA	Maceration	Bleach+EtOH
**OKM147**	*Paenibacillus polymyxa*	Bacteria	Hemp	Altair	Seedling	TSA	Maceration	Bleach+EtOH
**OKM148**	*Paenibacillus mobilis*	Bacteria	Hemp	Altair	Seedling	TSA	Maceration	Bleach+EtOH
**OKM149**	*Bacillus megaterium*	Bacteria	Hemp	Altair	Seedling	TSA	Maceration	Bleach+EtOH
**OKM150**	*Paenibacillus vulneris*	Bacteria	Hemp	Altair	Seedling	TSA	Maceration	Bleach+EtOH
**OKM151**	*Paenibacillus illinoisensis*	Bacteria	Hemp	Katani	Seedling	TSA	Maceration	Bleach+EtOH
**OKM153**	*Paenibacillus yunnanensis*	Bacteria	Hemp	Katani	Seedling	TSA	Maceration	Bleach+EtOH
**OKM154**	*Paenibacillus mobilis*	Bacteria	Hemp	Katani	Seedling	TSA	Maceration	Bleach+EtOH
**OKM155**	*Paenibacillus polymyxa*	Bacteria	Hemp	Katani	Seedling	TSA	Maceration	Bleach+EtOH
**OKM156**	*Paenibacillus illinoisensis*	Bacteria	Hemp	Katani	Seedling	TSA	Maceration	Bleach+EtOH
**OKM157**	*Paenibacillus mobilis*	Bacteria	Hemp	Grandi	Seedling	TSA	Maceration	Bleach+EtOH
**OKM158**	*Paenibacillus mobilis*	Bacteria	Hemp	Altair	Seedling	R2A	Maceration	Bleach+EtOH
**OKM159**	*Paenibacillus mobilis*	Bacteria	Hemp	Grandi	Seedling	TSA	Maceration	Bleach+EtOH
**OKM160**	*Paenibacillus polymyxa*	Bacteria	Hemp	Altair	Seedling	R2A	Maceration	Bleach+EtOH
**OKM161**	*Paenibacillus mobilis*	Bacteria	Hemp	Altair	Seedling	R2A	Maceration	Bleach+EtOH
**OKM162**	*Bacillus subtilis*	Bacteria	Hemp	Grandi	Seedling	TSA	Maceration	Bleach+EtOH
**OKM163**	*Bacillus subtilis*	Bacteria	Hemp	Altair	Seedling	R2A	Maceration	Bleach+EtOH
**OKM165**	*Paenibacillus mobilis*	Bacteria	Hemp	LKCSD	Seedling	R2A	Maceration	Bleach+EtOH
**OKM166**	*Paenibacillus mobilis*	Bacteria	Hemp	LKCSD	Seedling	R2A	Maceration	Bleach+EtOH
**OKM167**	*Paenibacillus mobilis*	Bacteria	Hemp	LKCSD	Seedling	TSA	Maceration	Bleach+EtOH
**OKM168**	*Paenibacillus mobilis*	Bacteria	Hemp	LKCSD	Seedling	TSA	Maceration	Bleach+EtOH
**OKM169**	*Bacillus subtilis*	Bacteria	Hemp	LKCSD	Seedling	TSA	Maceration	Bleach+EtOH
**OKM170**	*Paenibacillus polymyxa*	Bacteria	Hemp	FIN34	Seedling	TSA	Maceration	Bleach+EtOH
**OKM171**	*Paenibacillus mobilis*	Bacteria	Hemp	FIN34	Seedling	TSA	Maceration	Bleach+EtOH
**OKM172**	*Paenibacillus humicus*	Bacteria	Hemp	FIN34	Seedling	TSA	Maceration	Bleach+EtOH
**OKM173**	*Bacillus subtilis*	Bacteria	Hemp	FIN34	Seedling	TSA	Maceration	Bleach+EtOH
**OKM174**	*Bacillus megaterium*	Bacteria	Hemp	FIN34	Seedling	R2A	Maceration	Bleach+EtOH
**OKM175**	*Paenibacillus mobilis*	Bacteria	Hemp	FIN34	Seedling	R2A	Maceration	Bleach+EtOH
**OKM176**	*Paenibacillus sinopodophylli*	Bacteria	Hemp	FIN34	Seedling	R2A	Maceration	Bleach+EtOH
**OKM177**	*Paenibacillus terreus*	Bacteria	Hemp	FIN34	Seedling	R2A	Maceration	Bleach+EtOH
**OKM178**	*Bacillus zhangzhouensis*	Bacteria	Hemp	FIN34	Seedling	R2A	Maceration	Bleach+EtOH
**OKM180**	*Paenibacillus terreus*	Bacteria	Hemp	FIN34	Seedling	R2A	Maceration	Bleach+EtOH
**OKM181**	*Paenibacillus terreus*	Bacteria	Hemp	FIN34	Seedling	R2A	Maceration	Bleach+EtOH
**OKM182**	*Bacillus subtilis*	Bacteria	Hemp	FIN34	Seedling	R2A	Maceration	Bleach+EtOH
**OKM183**	*Paenibacillus pabuli*	Bacteria	Hemp	FIN34	Seedling	R2A	Maceration	Bleach+EtOH
**OKM184**	*Paenibacillus mobilis*	Bacteria	Hemp	Alyssa	Seedling	TSA	Maceration	Bleach+EtOH

**OKM185**	*Paenibacillus vulneris*	Bacteria	Hemp	Alyssa	Seedling	TSA	Maceration	Bleach+EtOH
**OKM186**	*Bacillus subtilis*	Bacteria	Hemp	Alyssa	Seedling	TSA	Maceration	Bleach+EtOH
**OKM187**	*Paenibacillus mobilis*	Bacteria	Hemp	Alyssa	Seedling	TSA	Maceration	Bleach+EtOH
**OKM188**	*Paenibacillus senegalensis*	Bacteria	Hemp	Alyssa	Seedling	TSA	Maceration	Bleach+EtOH
**OKM189**	*Bacillus zhangzhouensis*	Bacteria	Hemp	Alyssa	Seedling	TSA	Maceration	Bleach+EtOH
**OKM190**	*Paenibacillus mobilis*	Bacteria	Hemp	Alyssa	Seedling	TSA	Maceration	Bleach+EtOH
**OKM191**	*Paenibacillus azotifigens*	Bacteria	Hemp	Alyssa	Seedling	TSA	Maceration	Bleach+EtOH
**OKM192**	*Bacillus/Peribacillus simplex*	Bacteria	Hemp	Alyssa	Seedling	R2A	Maceration	Bleach+EtOH
**OKM193**	*Bacillus zhangzhouensis*	Bacteria	Hemp	Alyssa	Seedling	R2A	Maceration	Bleach+EtOH
**OKM194**	*Bacillus/Peribacillus simplex*	Bacteria	Hemp	Alyssa	Seedling	R2A	Maceration	Bleach+EtOH
**OKM195**	*Paenibacillus taohuashanense*	Bacteria	Hemp	Alyssa	Seedling	R2A	Maceration	Bleach+EtOH
**OKM196**	*Bacillus subtilis*	Bacteria	Hemp	FIN34	Seedling	TSA	Maceration	Bleach+EtOH
**OKM197**	*Bacillus/Peribacillus simplex*	Bacteria	Hemp	FIN34	Seedling	TSA	Maceration	Bleach+EtOH
**OKM198**	*Psychrobacter pulmonis*	Bacteria	Marijuana	God's Bud	Seedling	TSA	Maceration	Bleach+EtOH
**OKM199**	*Bacillus megaterium*	Bacteria	Hemp	X59 Gen2	Seedling	R2A	Maceration	Bleach+EtOH
**OKM200**	*Paenibacillus taohuashanense*	Bacteria	Hemp	X59 Gen2	Seedling	R2A	Maceration	Bleach+EtOH
**OKM201**	*Bacillus wiedmannii*	Bacteria	Hemp	X59 Gen2	Seedling	R2A	Maceration	Bleach+EtOH
**OKM202**	*Paenibacillus mobilis*	Bacteria	Hemp	X59 Gen2	Seedling	R2A	Maceration	Bleach+EtOH
**OKM203**	*Paenibacillus pabuli*	Bacteria	Hemp	X59 Gen2	Seedling	R2A	Maceration	Bleach+EtOH
**OKM204**	*Bacillus ginsengihumi*	Bacteria	Hemp	X59 Gen2	Seedling	R2A	Maceration	Bleach+EtOH
**OKM205**	*Paenibacillus mobilis*	Bacteria	Hemp	X59 Gen2	Seedling	R2A	Maceration	Bleach+EtOH
**OKM339**	*Bacillus megaterium*	Bacteria	Flax	Bethune-muc	Seedling	TSA	Maceration	Bleach+EtOH
**OKM340**	*Bacillus megaterium*	Bacteria	Flax	Bethune-muc	Seedling	R2A	Maceration	Bleach+EtOH
**PM17**	Penicillium brevicompactum	Fungi	Marijuana	God's Bud	Seedling	PDA	Maceration	Bleach+EtOH
**PM35**	Alternaria destruens	Fungi	Hemp	Altair	Seedling	PDA	Seed germination	
**PM36**	Penicillium robsamsonii	Fungi	Hemp	Altair	Seedling	PDA	Seed germination	
**PM37**	Penicillium aurantiogriseum	Fungi	Hemp	Altair	Seedling	PDA	Seed germination	
**PM38**	Cladosporium crousii	Fungi	Marijuana	BC Big Bud	Seedling	PDA	Seed germination	
**PM39**	Apiospora marii	Fungi	Marijuana	BC Big Bud	Seedling	PDA	Seed germination	
**PM40**	Aspergillus fumigatus	Fungi	Hemp	CAN3797	Seedling	PDA	Seed germination	
**PM41**	Aspergillus fumigatus	Fungi	Hemp	CAN3797	Seedling	PDA	Seed germination	
**PM42**	Cladosporium crousii	Fungi	Hemp	CAN3797	Seedling	PDA	Seed germination	
**PM43**	Chaetomium ovatoascomatis	Fungi	Hemp	Fin34	Seedling	PDA	Seed germination	
**PM45**	Aspergillus cibarius	Fungi	Hemp	Fin34	Seedling	PDA	Seed germination	
**PM46**	Alternaria destruens	Fungi	Hemp	Grandi	Seedling	PDA	Seed germination	
**PM47**	Alternaria destruens	Fungi	Hemp	Grandi	Seedling	PDA	Seed germination	
**PM48**	Alternaria destruens	Fungi	Hemp	Grandi	Seedling	PDA	Seed germination	
**PM49**	Rhizopus arrhizus	Fungi	Hemp	Katani	Seedling	PDA	Seed germination	
**PM50**	Penicillium citrinum	Fungi	Hemp	LKCSD	Seedling	PDA	Seed germination	
**PM51**	Aspergillus tubingensis	Fungi	Marijuana	God's Bud	Seedling	PDA	Seed germination	
**PM52**	Penicillium lanosocoeruleum	Fungi	Marijuana	God's Bud	Seedling	PDA	Seed germination	
**PM53**	Aureobasidium pullulans	Fungi	Hemp	X-59	Seedling	PDA	Seed germination	
**PM54**	Hormonema macrosporum	Fungi	Hemp	X-59	Seedling	PDA	Seed germination	
**PM55**	Hormonema macrosporum	Fungi	Hemp	X-59	Seedling	PDA	Seed germination	
**PM56**	Condenascus tortuosus	Fungi	Hemp	X59 Gen2	Seedling	PDA	Seed germination	
**PM60**	Fusarium fujikuroi	Fungi	hemp	VIR 577	Seedling	PDA	Seed germination	
**PM61**	Penicillium citrinum	Fungi	hemp	VIR 577	Seedling	PDA	Seed germination	
**PM62**	Aspergillus tubingensis	Fungi	hemp	VIR 577	Seedling	PDA	Seed germination	
**PM63**	Aspergillus ochraceus	Fungi	hemp	VIR 577	Seedling	PDA	Seed germination	

Figure S1. 16S Phylogenetic Tree of Culturable Seed-borne Cannabis Seedling Endophytes Across Hemp X59 Generations.

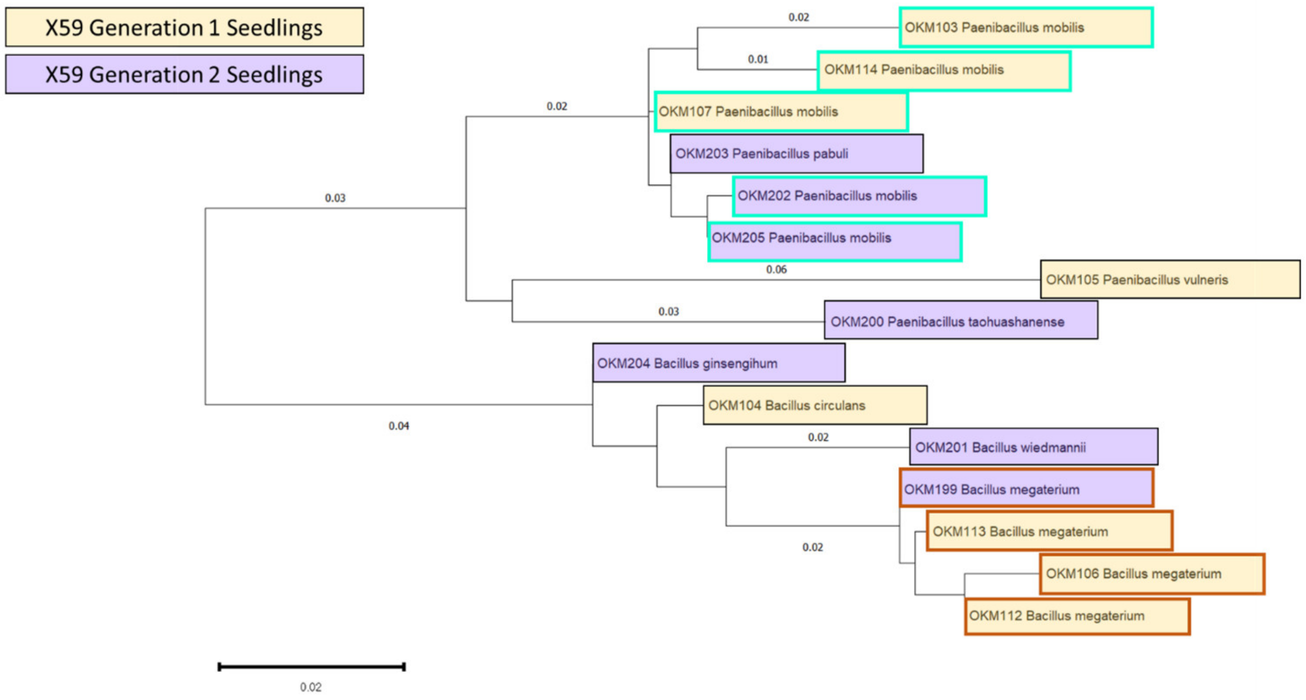

Strains of *Bacillus megaterium* and *Paenibacillus mobilis* were cultured from two successive generations of Hemp X59 grown to seed. Generation-1 was grown outdoors in soil in Vegreville, AB, Generation-2 was grown indoors in Sunshine Mix in Kelowna, BC. *P. mobilis* isolates are outlined in teal. *B. megaterium* isolates are outlined in orange. Amplicons were aligned using MUSCLE algorithim prior to nearest-neighbor phylogenetic tree was contruction using Tamura-Nei model on MEGA software (Version 11.0.11).

Figure S2. Altair Seed-Associated Fungi.

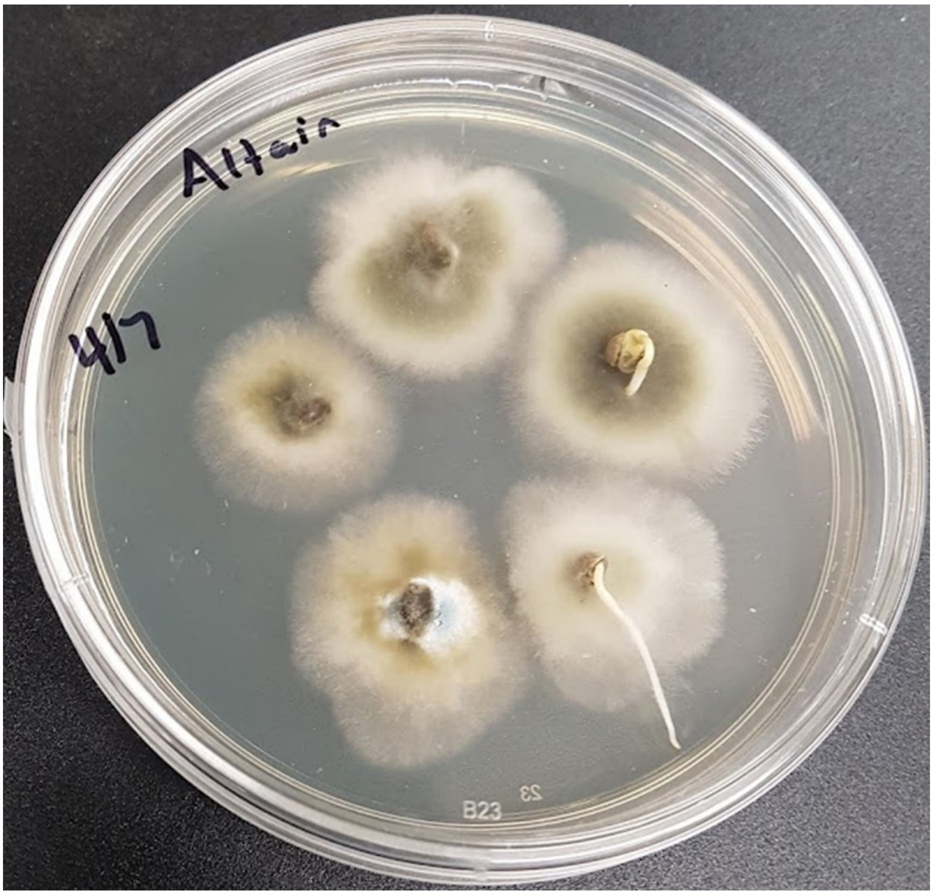

Alternaria and *Penicillium* growth on germinating Altair hemp seeds 4 days after planting on PDA.
